# Supplementary material for: Quantifying resilience and the risk of regime shifts under strong correlated noise
Source: PNAS Nexus. 2022 Dec 24;2(2):pgac296. doi: 10.1093/pnasnexus/pgac296 (PMC9896148; doi:10.1093/pnasnexus/pgac296)
Supplement: pgac296_Supplemental_File [file pgac296_supplemental_file.pdf]

PNAS Nexus, 2023, **2**, 1–8
https://doi.org/10.1093/pnasnexus/pgac296
Advance access publication date: 24 December 2022
Supplementary Information

SUPPLEMENTARY INFORMATION

Supplementary Information for
Quantifying resilience and the risk of regime shifts under strong correlated noise

Martin Heßler[a,b] and Oliver Kamps[b]

[a]Institute for Theoretical Physics, Westphalian Wilhelms-University Münster, Wilhelm-Klemm-Straße 9, 48149, North Rhine-Westphalia, Germany and [b]Center for Nonlinear Science, Westphalian Wilhelms-University Münster, Corrensstraße 2, 48149, North Rhine-Westphalia, Germany
Received on 19 April 2022; accepted on 20 December 2022

Corresponding author: Martin Heßler
E-mail: m_hess23@uni-muenster.de

This pdf includes:

- Supplementary text
- Tables S1 to S4
- Figures S1 to S6
- SI references

Other supplementary materials for this manuscript include the following:

- Data and simulation codes can be found at `https://github.com/MartinHessler/Quantifying_resilience_under_realistic_noise`.

© The Author 2023. Published by Oxford University Press. All rights reserved. For permissions, please e-mail: journals.permissions@oup.com

Supplementary Information Text

Negligible influence of the trend component

In order to isolate the influence of the seasonality of the data regarding the leading indicator estimation, the Bayesian model comparison is repeated for the measures computed on the datasets detrended by subtracting a Gaussian kernel smoothing with kernelwidth of 150. The smoothing is performed by the Python function *scipy.ndimage.filters.gaussian_filter* [8]. The results considering a linear model $\mathcal{M}_1$ with positive slope are presented in table S1 and complemented by the Bayes factors of the skewness based on a linear model $\mathcal{M}_1$ with negative slope in table S2. By comparing the tables in the main article without any preprocessing (cf. main article, table 1) to the detrended ones shown here, we can deduce that the influence of the trend component of the time series is negligible in contrast to its seasonality.

Bayes factors of skewness assuming a linear model with negative slope

For completeness of the presented analysis, the Bayesian model comparison of the skewness is also performed assuming negative slopes for the linear model $\mathcal{M}_1$ without and with deseasonalization. The results, shown in table S3 and S4, confirm the calculations stated in the main article (cf. main article, tables 1 and 2).

Leading indicator calculation and exclusion of the kurtosis from further analysis

Furthermore, we present the statistical measures of each considered case for inspection by eye in the figures S1, S2 and S3 without any preprocessing, with deseasonalization and with detrending, respectively. Most of the cases under study do not exhibit a clear trend of the kurtosis which is therefore excluded from further analysis in the main article. In the end, the generally late increase of the standard deviation as well as the improved clear trends of autocorrelation and skewness in the deseasonalized cases is underlined by the results of figures S1, S2 and S3.

Analytical drift slope

The drift slope of the planktivore F-component in planktivore F-direction is analytically computed for a quantitative comparison in figure 3 of the main article. One example is shown in figure S4. The time series realisations of the model are plugged into the analytical form of

$$\frac{\partial}{\partial F}\left(\frac{\mathrm{d}F}{\mathrm{d}t}\right) = -D_{\mathrm{F}} - c_{\mathrm{FA}} \cdot A \tag{1}$$

to compute the drift slope. The result for the pink noise case with noise level $\sigma = 4.5$ is shown as the blue-shaded line in figure S4. A Gaussian kernel smoothing with width 50 via *scipy.ndimage.filters.gaussian_filter* [8] is used to get an impression of the average evolution of the drift slope without seasonality effects. The corresponding smoothed drift slope is presented as orange line in figure S4.

Direct drift slope estimation

A simplistic way to estimate the drift is proposed in [2, 3]. Briefly summarized the data is binned, the average drift per bin is computed and a polynomial of order one or of order three is fitted to the data. The derivative of the resulting parameterization is computed in the fixed point that is estimated as the average time series value per data window. The results of that *direct drift slope estimation* applied to the investigated realisations of the ecological model without and with deseasonalization are shown for both a linear fit and a polynomial fit of order three in the figures S5 and S6, respectively. In contrast to the Bayesian drift slope estimation (cf. main article, figure 3) the direct drift slope estimation does not provide quantitatively reliable results of the drift slope in the case of weak white noise as shown in the figures S5 (A) and S6 (A). Furthermore, the direct drift slope estimates are much more noisy in general, probably due to the small amount of data per bin in a rolling window approach, and do not replicate the analytical drift slope indicated by the black dotted lines as precise as the Bayesian drift slope estimates in the white noise cases in the figures S5 (B,C), which complicates their interpretation even more due to the absence of intrinsic uncertainty estimates. In the colored noise cases in figure S5 (D-I) the stability of the direct drift slope estimation via a polynomial breaks down completely. Its linear fit pendant yields at least a qualitative trend measure of the ongoing destabilization processes, but deviates significantly more from the analytical drift slopes as the corresponding estimates of the Bayesian drift-diffusion estimation method (cf. main article, figure 3).
In order to give a comprehensive picture of the limits of the direct slope estimation procedure in comparison to the Bayesian drift slope estimation the analysis of the model realisations is repeated for the deseasonalized versions of the main article in figure S6. The polynomial fits do not yield reasonable results and the linear counterparts tend to be less

Table S1: Summary of the Bayes factors comparing a linear model $\mathcal{M}_1$ with positive slope to a constant model $\mathcal{M}_2$ for the drift slope ζ, the AR1, the std $\hat{\sigma}$ and the skewness γ with detrending of the data. The kurtosis is excluded because of its non-monotone behaviour. Green tiles mark a $BF_{12} > 100$ [5, 6, 4] which is the threshold for a significant leading indicator trend. Grey tiles mark insignificant results. The constant model $\mathcal{M}_2$ is never preferred in the analysis. Infinite Bayes factors result from one model with evidence zero which leads to preferring the finite evidence model. Apart from slightly different values the significance does not change compared to the Bayes factors of the original data (cf. main article, table 1). This confirms the conclusion that the seasonality has a predominant importance for the calculation of the standard leading indicators in the considered ecological model.

	white noise ($P_{\text{white}} \approx 7.5$)			pink noise ($P_{\text{pink}} \approx 2 \cdot P_{\text{white}}$)			red noise ($P_{\text{red}} \approx P_{\text{pink}}$)		
noise level	$\sigma = 0.1$	$\sigma = 2.2$	$\sigma = 4.5$	$\sigma = 0.1$	$\sigma = 2.2$	$\sigma = 4.5$	$\sigma = 0.1$	$\sigma = 2.2$	$\sigma = 4.5$
indicator $\mathcal{I}$	BF_{12}	BF_{12}	BF_{12}	BF_{12}	BF_{12}	BF_{12}	BF_{12}	BF_{12}	BF_{12}
	BF_{21}	BF_{21}	BF_{21}	BF_{21}	BF_{21}	BF_{21}	BF_{21}	BF_{21}	BF_{21}
slope ζ	∞	∞	∞	∞	∞	∞	∞	$2.3 \cdot 10^{140}$	$1.5 \cdot 10^{145}$
	0	0	0	0	0	0	0	$4.4 \cdot 10^{-141}$	$6.7 \cdot 10^{-146}$
AR1	14	$7.6 \cdot 10^{7}$	$2.4 \cdot 10^{7}$	11	11	9.7	11	4.8	1.8
	$7.0 \cdot 10^{-2}$	$1.3 \cdot 10^{-8}$	$4.1 \cdot 10^{-8}$	$9.0 \cdot 10^{-2}$	$9.5 \cdot 10^{-2}$	0.10	$9.0 \cdot 10^{-2}$	0.21	0.55
std $\hat{\sigma}$	15	15	36	15	15	28	15	9.3	$2.4 \cdot 10^{3}$
	$6.6 \cdot 10^{-2}$	$6.9 \cdot 10^{-2}$	$2.8 \cdot 10^{-2}$	$6.8 \cdot 10^{-2}$	$6.9 \cdot 10^{-2}$	$3.5 \cdot 10^{-2}$	$6.7 \cdot 10^{-2}$	1.1	$4.1 \cdot 10^{-4}$
skewness γ	$6.5 \cdot 10^{6}$	$5.4 \cdot 10^{5}$	0.67	$1.2 \cdot 10^{7}$	$9.1 \cdot 10^{5}$	$2.8 \cdot 10^{7}$	$1.0 \cdot 10^{7}$	97	270
	$1.5 \cdot 10^{-7}$	$1.9 \cdot 10^{-6}$	1.5	$8.4 \cdot 10^{-8}$	$1.1 \cdot 10^{-6}$	$3.6 \cdot 10^{-8}$	$9.8 \cdot 10^{-8}$	$1.0 \cdot 10^{-2}$	$3.7 \cdot 10^{-3}$

Table S2: Summary of the Bayes factors comparing a linear model $\mathcal{M}_1$ with negative slope to a constant model $\mathcal{M}_2$ for the skewness γ for various noise types and levels with detrending of the data. Green tiles mark a $BF_{12} > 100$ which is the threshold for a significant leading indicator trend. Grey tiles mark insignificant results.

	white noise ($P_{\text{white}} \approx 7.5$)			pink noise ($P_{\text{pink}} \approx 2 \cdot P_{\text{white}}$)			red noise ($P_{\text{red}} \approx P_{\text{pink}}$)		
noise level	$\sigma = 0.1$	$\sigma = 2.2$	$\sigma = 4.5$	$\sigma = 0.1$	$\sigma = 2.2$	$\sigma = 4.5$	$\sigma = 0.1$	$\sigma = 2.2$	$\sigma = 4.5$
indicator $\mathcal{I}$	BF_{12}	BF_{12}	BF_{12}	BF_{12}	BF_{12}	BF_{12}	BF_{12}	BF_{12}	BF_{12}
	BF_{21}	BF_{21}	BF_{21}	BF_{21}	BF_{21}	BF_{21}	BF_{21}	BF_{21}	BF_{21}
skewness γ	$1.7 \cdot 10^{-2}$	$2.0 \cdot 10^{-2}$	0.14	$1.5 \cdot 10^{-2}$	$1.8 \cdot 10^{-2}$	$1.5 \cdot 10^{-2}$	$1.5 \cdot 10^{-2}$	$3.7 \cdot 10^{-2}$	$2.0 \cdot 10^{-2}$
	60	50	6.8	67	55	67	67	27	49

Table S3: Bayesian model comparison with a linear model $\mathcal{M}_1$ with negative slope and a constant model $\mathcal{M}_2$ for the skewness computed on the data without deseasonalization or detrending. Grey tiles mark insignificant results, i.e. $BF_{ij} \leq 100$.

	white noise ($P_{\text{white}} \approx 7.5$)			pink noise ($P_{\text{pink}} \approx 2 \cdot P_{\text{white}}$)			red noise ($P_{\text{red}} \approx P_{\text{pink}}$)		
noise level	$\sigma = 0.1$	$\sigma = 2.2$	$\sigma = 4.5$	$\sigma = 0.1$	$\sigma = 2.2$	$\sigma = 4.5$	$\sigma = 0.1$	$\sigma = 2.2$	$\sigma = 4.5$
indicator $\mathcal{I}$	BF_{12}	BF_{12}	BF_{12}	BF_{12}	BF_{12}	BF_{12}	BF_{12}	BF_{12}	BF_{12}
	BF_{21}	BF_{21}	BF_{21}	BF_{21}	BF_{21}	BF_{21}	BF_{21}	BF_{21}	BF_{21}
skewness γ	$1.5 \cdot 10^{-2}$	$1.9 \cdot 10^{-2}$	0.12	$1.4 \cdot 10^{-2}$	$1.9 \cdot 10^{-2}$	$1.2 \cdot 10^{-2}$	$1.4 \cdot 10^{-2}$	$3.6 \cdot 10^{-2}$	$1.5 \cdot 10^{-2}$
	65	52	8.6	70	54	84	70	28	67

stable in general. In conclusion the direct drift slope estimation is only a reasonable alternative in a very limited number of cases.

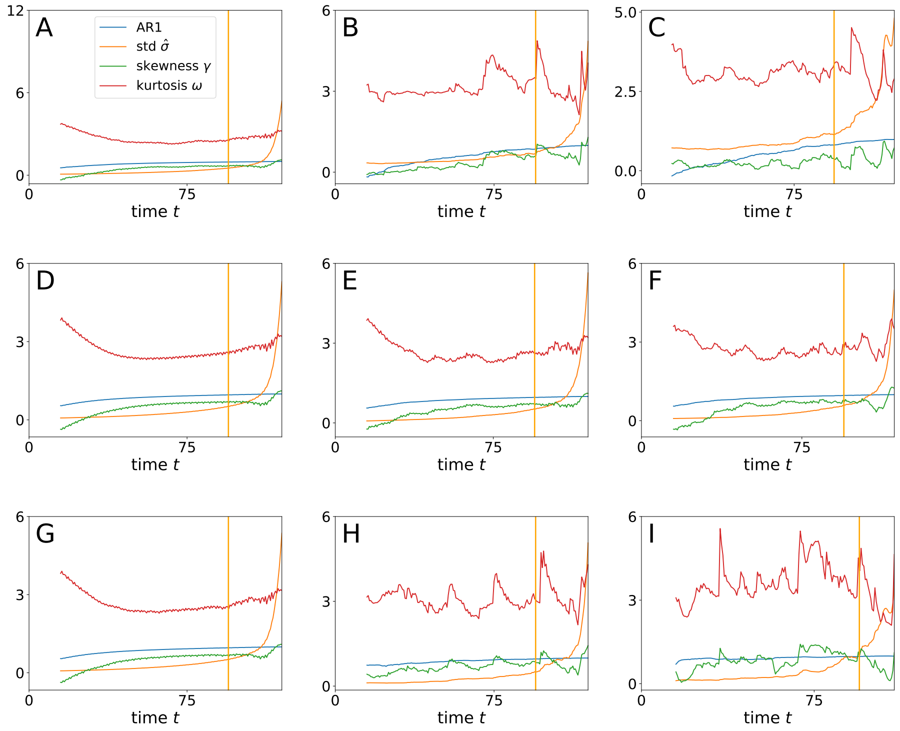

Fig. S1. Statistical measures calculated for the datasets without preprocessing, i.e. without deseasonalization or detrending. (A-C) White noise cases, (D-F) pink noise cases and (G-I) red noise cases with increasing noise levels $\sigma = \{0.1, 2.2, 4.5\}$. The solid orange vertical line marks the "point of no return" up to which the data is used for the Bayesian model comparison in analogy to [7] and [1].

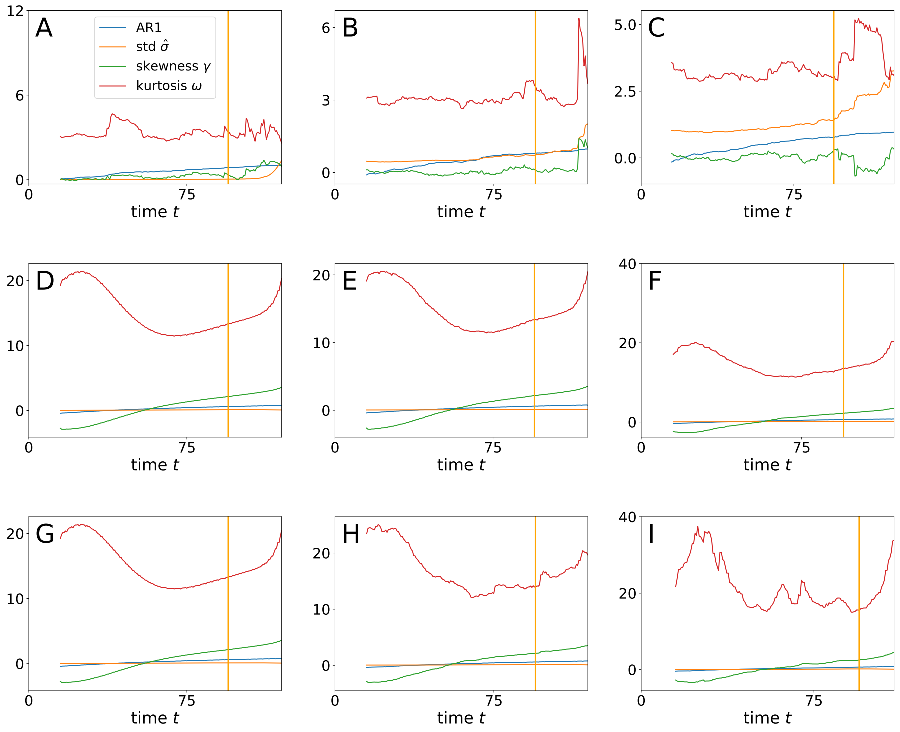

Fig. S2. Statistical measures calculated for the datasets with deseasonalization. (A-C) White noise cases, (D-F) pink noise cases and (G-I) red noise cases with increasing noise levels $\sigma = \{0.1, 2.2, 4.5\}$. The solid orange vertical line marks the "point of no return" up to which the data is used for the Bayesian model comparison in analogy to [7] and [1].

Table S4: Bayesian model comparison with a linear model $\mathcal{M}_1$ with negative slope and a constant model $\mathcal{M}_2$ for the skewness computed on the data with deseasonalization. Orange tiles mark a $BF_{21} > 100$ which is the threshold for preferring the constant model. Grey tiles mark insignificant results.

	white noise ($P_{\text{white}} \approx 7.5$)			pink noise ($P_{\text{pink}} \approx 2 \cdot P_{\text{white}}$)			red noise ($P_{\text{red}} \approx P_{\text{pink}}$)		
noise level	$\sigma = 0.1$	$\sigma = 2.2$	$\sigma = 4.5$	$\sigma = 0.1$	$\sigma = 2.2$	$\sigma = 4.5$	$\sigma = 0.1$	$\sigma = 2.2$	$\sigma = 4.5$
indicator $\mathcal{I}$	BF_{12}	BF_{12}	BF_{12}	BF_{12}	BF_{12}	BF_{12}	BF_{12}	BF_{12}	BF_{12}
	BF_{21}	BF_{21}	BF_{21}	BF_{21}	BF_{21}	BF_{21}	BF_{21}	BF_{21}	BF_{21}
skewness γ	$5.3 \cdot 10^{-2}$	0.16	0.19	$4.7 \cdot 10^{-3}$	$4.5 \cdot 10^{-3}$	$5.4 \cdot 10^{-3}$	$4.7 \cdot 10^{-3}$	$5.4 \cdot 10^{-3}$	$4.8 \cdot 10^{-3}$
	19	6.1	5.2	214	222	187	210	180	210

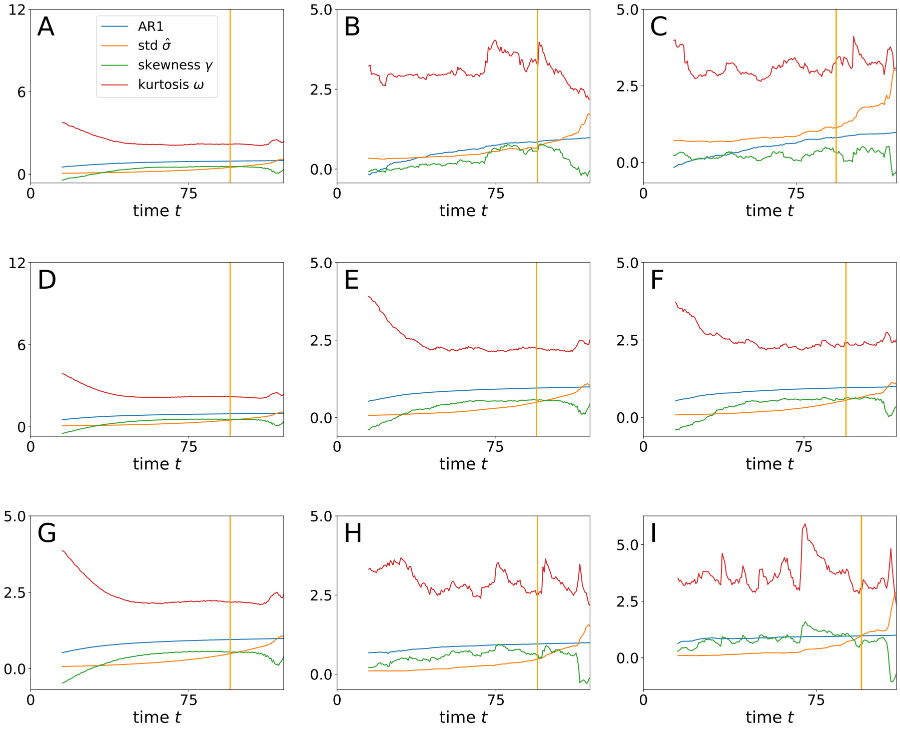

Fig. S3. Statistical measures calculated for the datasets with detrending. (A-C) White noise cases, (D-F) pink noise cases and (G-I) red noise cases with increasing noise levels $\sigma = \{0.1, 2.2, 4.5\}$. The detrending is performed by subtracting a Gaussian kernel smoothing with kernelwidth of 150 from the original data. The Python function *scipy.ndimage.filters.gaussian_filter* [8] is used for this procedure. The solid orange vertical line marks the "point of no return" up to which the data is used for the Bayesian model comparison in analogy to [7] and [1].

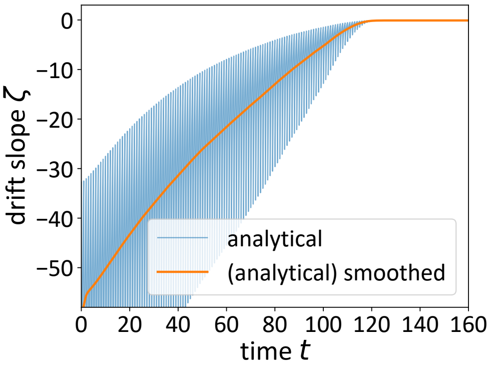

Fig. S4. Analytical slopes of the planktivore F-component in planktivore F-direction (blue line) of the pink noise case with noise level $\sigma = 4.5$. The seasonal component is smoothed via a Gaussian kernel smoothing with width 50 via *scipy.ndimage.filters.gaussian_filter* [8] as indicated by the orange line.

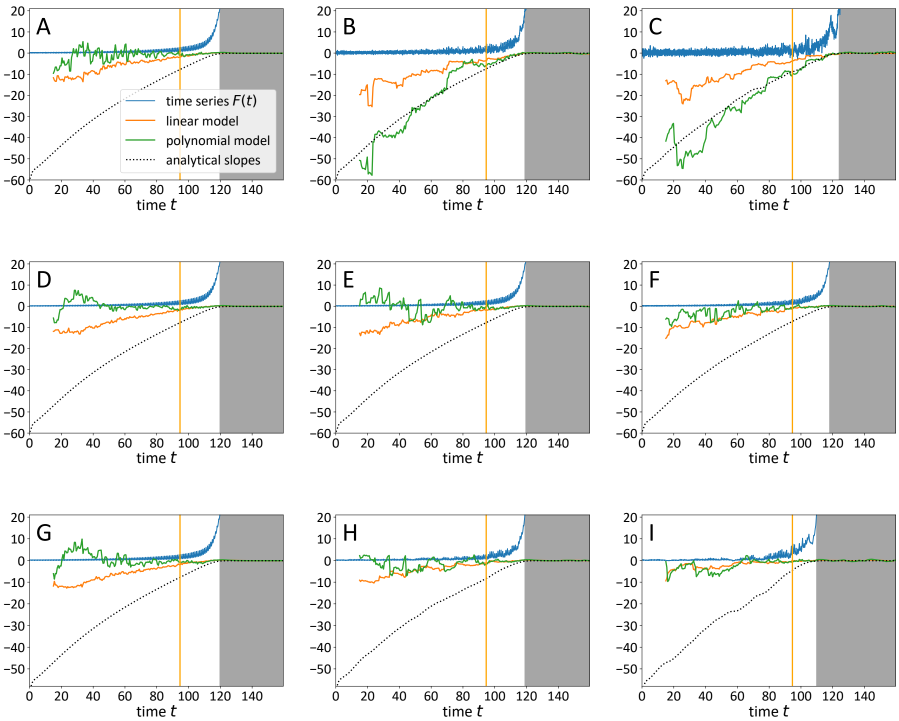

Fig. S5. The results of the direct drift slope estimation procedure discussed in subsection 1.5 are presented in the figure. The results of the first and the third order polynomial fit are indicated by the orange and green lines, respectively. The analysed time series are shown in blue. The analytical drift slope of the planktivores in planktivore F-direction is shown as black dotted line. The point of no return as defined in the main article is marked by the vertical orange line and the post-transition zone is signed by the grey shaded area. It is subjectively defined to guide the eye as the time at which the planktivore F-population exceeds 21 individuals for the first time. In contrast to the Bayesian drift slope estimates of the main article (cf. main article, figure 3) the direct estimation results deviate from the analytical values in the weak white noise case (A). In general the results are more erratic than the Bayesian estimates and do not yield intrinsic uncertainty estimates which makes them more difficult to interpret especially in an on-line approach. In the colored noise cases (D-I) the polynomial estimates become unstable and the linear ones deviate much more from the analytical values than observed for the Bayesian estimates in the main article.

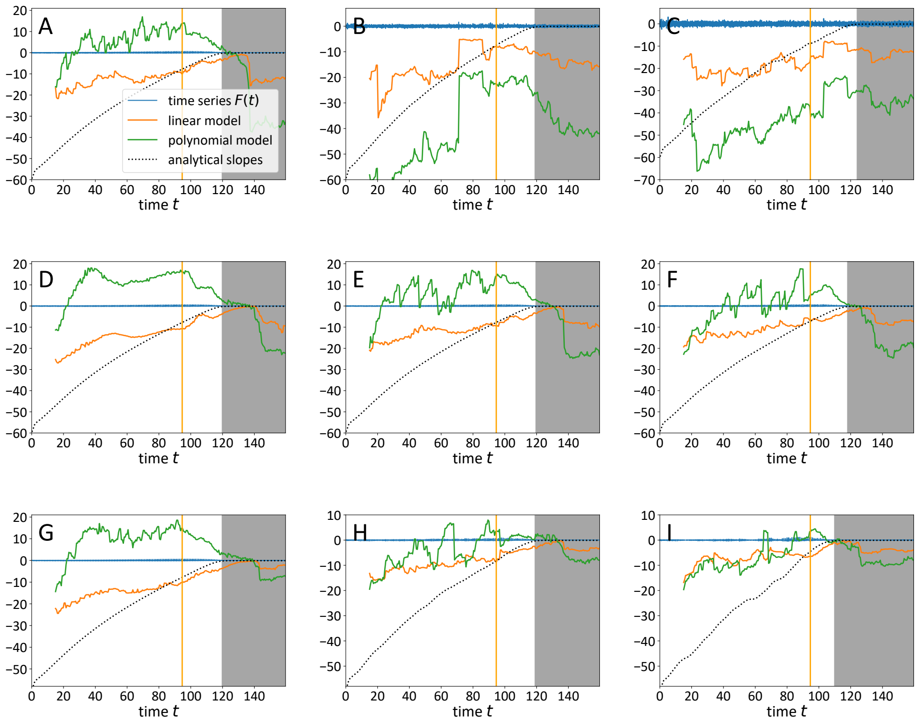

Fig. S6. Same as figure S5, but for the deseasonalized versions of the time series. The polynomial estimates yield unreasonable results, whereas the linear ones tend to be less stable.

SI references

1. Reinette Biggs, Stephen R. Carpenter, and William A. Brock. Turning back from the brink: Detecting an impending regime shift in time to avert it. *Proceedings of the National Academy of Sciences*, 106(3):826–831, jan 2009.
2. R. Friedrich and J. Peinke. Description of a turbulent cascade by a fokker-planck equation. *Phys. Rev. Lett.*, 78:863–866, Feb 1997.
3. R. Friedrich, S. Siegert, J. Peinke, St. Lück, M. Siefert, M. Lindemann, J. Raethjen, G. Deuschl, and G. Pfister. Extracting model equations from experimental data. *Physics Letters A*, 271(3):217–222, jun 2000.
4. Harold Jeffreys. *Theory of Probability.* OUP Oxford, August 1998.
5. Robert E. Kass and Adrian E. Raftery. Bayes factors. *Journal of the American Statistical Association*, 90(430):773–795, jun 1995.
6. M.D. Lee and E.J. Wagenmakers. *Bayesian Cognitive Modeling: A Practical Course.* Bayesian Cognitive Modeling: A Practical Course. Cambridge University Press, 2014.
7. Charles T. Perretti and Stephan B. Munch. Regime shift indicators fail under noise levels commonly observed in ecological systems. *Ecological Applications*, 22(6):1772–1779, sep 2012.
8. Pauli Virtanen, Ralf Gommers, Travis E. Oliphant, Matt Haberland, Tyler Reddy, David Cournapeau, Evgeni Burovski, Pearu Peterson, Warren Weckesser, Jonathan Bright, Stéfan J. van der Walt, Matthew Brett, Joshua Wilson, K. Jarrod Millman, Nikolay Mayorov, Andrew R. J. Nelson, Eric Jones, Robert Kern, Eric Larson, C J Carey, İlhan Polat, Yu Feng, Eric W. Moore, Jake VanderPlas, Denis Laxalde, Josef Perktold, Robert Cimrman, Ian Henriksen, E. A. Quintero, Charles R. Harris, Anne M. Archibald, Antônio H. Ribeiro, Fabian Pedregosa, Paul van Mulbregt, and SciPy 1.0 Contributors. SciPy 1.0: Fundamental Algorithms for Scientific Computing in Python. *Nature Methods*, 17:261–272, 2020.
